# Hidden blood loss of total knee arthroplasty in hemophilia arthritis: an analysis of influencing factors

**DOI:** 10.1186/s12891-022-05535-y

**Published:** 2022-06-17

**Authors:** Shao Ning Shen, Dong Xiao Wu, Shuai Jie Lv, Pei Jian Tong

**Affiliations:** The First Clinical College of Zhejiang Chinese Medical University, The First Affiliated Hospital of Zhejiang Chinese Medical University, 548# BinWen Road, HangZhou, ZheJiang Province People’s Republic of China

**Keywords:** Hemophilia A, Total joint arthroplasty, Hidden blood loss, Influential factor

## Abstract

**Background:**

Total knee arthroplasty is the leading way to treat hemophilia arthritis. At present, there is a lack of research on the influencing factors of blood loss in total knee arthroplasty for hemophilia arthritis. This study comprehensively explores the definite factors affecting the hidden blood loss in total knee arthroplasty for hemophilia patients.

**Materials and methods:**

Ninety-two hemophilia A patients who underwent total knee arthroplasty in our center were included. Demographics, laboratory data, surgical data, and complications were collected. The Gross equation and Sehat equation were used to calculate the estimated value of hidden blood loss. Multivariate linear regression analysis was used to determine the influencing factors of hidden blood loss.

**Result:**

The hidden blood loss of hemophilia A patients undergoing total knee arthroplasty was 1069.51 ± 341.99 mL, and the age was positively correlated with the hidden blood loss (*P* < 0.001), while tranexamic acid, FVIII prophylaxis, and incremental invivo recovery were negatively correlated with the hidden blood loss (*P* < 0.001, *P* = 0.008, *P* = 0.017).

**Conclusion:**

Elderly patients have a greater risk of blood loss, and additional preventive measures can be appropriately added. Intraoperative intra-articular injection of tranexamic acid is recommended to reduce hidden blood loss, FVIII prophylaxis is recommended for every patient. We recommend that all HA patients measure the incremental invivo recovery and develop a personalized infusion regimen of coagulation factor.

## Background

Hemophilic knee arthritis is a common complication of hemophilia A (HA). Total knee arthroplasty (TKA) is the main treatment of hemophilic knee arthritis, which has made remarkable effects on relieving pain and improving function [1–3]. However, hidden blood loss after the operation is still a concern of clinicians. Even if the clotting factor concentrates (CFCS) have been fully used in the perioperative period to reach the required level of FactorVIII (FVIII) in vivo [4], but many studies have showed that the blood loss of HA patients is much higher than that of ordinary patients [5, 6], which leads to more blood transfusion, higher hospitalization expenses, and worse recovery effect. A reasonable blood management strategy is very important, because hemophilia is easy to bleed. Determining the influencing factors of blood loss is an important step to establishing an effective blood management strategy.

However, there is still a lack of research on the influencing factors of TKA hidden blood loss in HA patients. This study included 92 HA patients who underwent unilateral TKA in our center from January 2015 to October 2020. Multivariate linear regression was used to explore the influencing factors of postoperative hidden blood loss, including age, Tranexamic acid (TXA), FVIII prophylaxis, incremental invivo recovery (IVR) etc. The purpose of this study is to comprehensively explore the exact factors influencing the hidden blood loss of HA patients undergoing total knee arthroplasty.

## Data and methods

### Ethical review

The clinical data of HA patients who underwent TKA in the Orthopedic Center of the First Affiliated Hospital of Zhejiang Chinese Medicine University from January 2015 to October 2020 were retrospectively collected and analyzed. This study was approved by the Ethics Review Committee of the First Affiliated Hospital of Zhejiang Chinese Medicine University and obtained the unique identification number of research registration (the research registration number is 2020047). Each patient signed a written informed consent form.

### Inclusion and exclusion criteria

The inclusion criteria include: ① A clear history of hemophilia A and the knee joint lesions meet the “Improved Arnold-Hilgartner Classification of Joint Lesions” Class IV diagnostic criteria [7]. ② One-stage unilateral total knee arthroplasty was performed in the Orthopedic Center of the First Affiliated Hospital of Zhejiang Chinese Medicine University.

Exclusion criteria include ① Lack of information including demographics, Laboratory data, surgical data. ② With Other diseases including Acquired Immune Deficiency Syndrome (AIDS), tumor. ③ Joint infection after surgery. ④ The positive inhibitors and/or Classical IVR of FVIII is less than 66%.

A total of 114 TKAs were performed during the study period. Ninety-two TKAs were performed in 83 patients who were enrolled after applying the inclusion and exclusion criteria. Two cases with no surgical data, three cases with AIDS, one case with rectal tumors,two cases with Joint infection after surgery, fourteen cases whose Classical IVR of FVIII is less than 66% were excluded.

### Operation method

All the operations were performed by the same team in our center, and all patients were given general anesthesia and a tourniquet for the whole process. We adopted standardized methods: the median knee approach, with the incision length ranging from 10 cm to 15 cm, opened the patella, separated the soft tissue layer by layer, opened the joint cavity, exposed the femoral end and tibial plateau, performed tibia and femur osteotomy sequentially, and deployed the prosthesis sample to determine the suitable prosthesis model. All the prosthesis were Posterior stabilized prosthesis (Zimmer Inc. Warsaw, IN, USA). And then fixed with bone cement. At our center, TXA was not used in all HA patients undergoing TKA between January 2015 and February 2018. We speculated that tranexamic acid would benefit HA patients. Therefore, TXA was used in all HA patients undergoing TKA from March 2018 to October 2020. It can be divided into two groups according to whether TXA was injected into the joint cavity during the operation. Before the tourniquet was released, TXA solution consisting of 1gTXA and 50 ml normal saline was injected into the joint cavity in the user group, and then the joint cavity was closed. Without TXA, the joint cavity was closed directly. No drainage tube was placed in all patients during the operation.

### Perioperative management

After admission, the level of FVIII was measured for the patients, then 2500 international units (IU) of CFCS (Bayer HealthCare LLC. Leverkusen, Germany). was injected into the patients experimentally, and 2500 IU was divided by the bodyweight to obtain the infusion dose per kilogram of body weight (iu·kg^− 1^). Re-measure the level of coagulation factors in patients at 1 hour, 2 hours, and 8 hours after injection to obtain the peak level and valley level of coagulation factors, Calculating the Classical IVR according to the formula: Classical IVR [8] = [the increase of actual coagulation factor (%or iu·dl^− 1^/the increase of expected coagulation factor (%or iu·dl^− 1^) × 100%. The expected increase of coagulation factor (% or iu·dl^− 1^) = infusion dose per kilogram of body weight (iu·kg^− 1^) × 2 (iu·dl^− 1^/iu·kg^− 1^). The Classical IVR was used as the basis of perioperative infusion dose of coagulation factors. According to the Expert Consensus on Perioperative Management of Hemophilia Orthopaedic Surgery in China [9] and Management Guide for Hemophilia [4], the FVIII level should be kept at 80–100% on the operation day, 60–80% on 1–3 days after the operation, 40–60% on 4–6 days after the operation and 30–50% on 7–14 days after the operation. All patients had no autologous blood transfusion. If the patient has HB < 70 g/L or HCT < 25%, it is considered as an indication that allogeneic blood transfusion is needed [10].

Patients had routine preoperative X-ray films of the anteroposterior and lateral position of the knee joint and full-length standing position of lower limbs, and Arnold-Higartner arthrosis classification was performed by the same professional radiologist [11]. Assess the patients according to the standard to determine the indications of total knee arthroplasty.

Cephalosporins antibiotics were routinely used to prevent infection 1 day before operation and 3 days after operation. On the first day after the operation, ice packs were applied around the knee joint for 24 hours to reduce postoperative bleeding. Considered for the particularity of hemophilia patients, we use intermittent lower limb pneumatic pumps to prevent Venous thromboembolism (VTE) of lower limbs instead of anticoagulants. From the second day after the operation, the same rehabilitation team in the center will perform postoperative rehabilitation training for patients, including passive and active knee joint movements, plantar flexion and extension movements, getting out of bed, and walking with walking auxiliary equipment.

### Data collection

From January 2015 to October 2020, 92 cases were included in the study. The recorded data included: sex, age, height, weight, preoperative Hematocrit (HCT), preoperative hemoglobin (HB), postoperative HCT, postoperative HB, FVIII peak level, FVIII valley level, TXA, FVIII prophylaxis, operation duration, intraoperative bleeding, blood transfusion.

### Quantitative and variable calculation methods

Sex, height, age, weight, HCT, HB, operation time, and FVIII peak level on the operation day were obtained directly from the medical records, in which the data of HCT and HB before operation were taken within 2 days before the operation, and those after the operation were taken at around 7 o’clock in the morning.

The preoperative BMI was calculated, BMI = weight (kg)/ height(m)^2^.

The level of FVIII valley is the level of FVIII measured after admission, and the peak level of FVIII is the level measured 1 hour after experimental infusion of FVIII. The Classical IVR is calculated according to the above formula, and the incremental IVR (IU·DL^− 1^/IU·kg^− 1^) = [FVIII peak level (% or IU·DL^− 1^-baseline FVIII level (% or IU·DL^− 1^)]/[infusion dose (IU·kg^− 1^)].

There are multiple time measurement points for postoperative HCT. Compare the HCT values of multiple time measurement points, take the lowest value as postoperative HCT, and use the Gross equation formula perfected by Gross. J B to calculate [12]. Gross equation specific calculation method: total blood loss = preoperative blood volume (PBV) × (preoperative HCT- postoperative HCT)/ (preoperative HCT+ postoperative HCT) × 2. PBV = kl × height(m)^^3^ + k2 × weight (kg) + k3.(male kl = 0.3669, k2 = 0.03219, k3 = 0.6041; Female kl = 0.3561, k2 = 0.03308, k3 = 0.1833). According to Sehat equation [13], Subtract the intraoperative blood loss from the total blood loss to get the hidden blood loss.

According to the operation records, the usage of TXA was obtained, including ① intra-articular infusion of 1gTXA + 50 ml normal saline; ② TXA is not used.

According to medical records or telephone follow-up, the FVIII prophylaxis of FVIII was obtained, including ① irregular FVIII prophylaxis of FVIII in the past year; ② In recent one year, there was a low dose regimen for prevention and treatment: 10 IU/kg per dose, which was given to hemophilia A patients twice times a week; ③ In recent one year, there was a middle dose regimen for prevention and treatment: 15 ~ 30 IU/kg per dose, which was given to hemophilia A patients three times a week; ④ In recent one year, there were high-dose FVIII prophylaxis s: 25 ~ 40 IU/kg per dose, which was given to hemophilia A patients three times a week.

According to the medical records, Infection with hepatitis B virus (HBV) was obtained, including ① No Infection with HBV; ② Infection with HBV.

### Multivariate linear regression analysis

Describe the statistical results, in which the continuous variables are shown as means±standard deviation; Classification is shown as a percentage. Continuity variables can be directly used as dependent variables of multi-factor linear regression analysis, and classified variables are assigned by numerical values: see Table 1 for specific treatment of variables. There are 19 factors as independent variables, Multi-factor linear regression analysis was carried out with the hidden blood loss after the operation as the dependent variable.

**Table 1:** Definition and assignment method of related factors affecting blood loss

Variable	Name	Definition and assignment
Y1	Total blood loss	ml
Y2	Intraoperative blood loss	ml
Y3	Hidden blood loss	ml
X1	BMI	kg/m^2^
X2	Age	year
X3	Sex	0 = male; 1 = female
X4	Total blood volume	ml
X5	TXA	0 = no TXA; 1 = TXA
X6	Regular FVIII prophylaxis	0 = untreated; 1 = low dose regimen; 2 = medium dose regimen; 3 = high-dose regimen;
X7	HBV	0 = no HBV; 1 = HBV
X8	Preoperative HB	g/L
X9	Postoperative HB	g/L
X10	Preoperative HCT	%
X11	Postoperative HCT	%
X12	FVIII peak level	% or IU·dL^−1^
X13	FVIII valley level	% or IU·dL^−1^
X14	Classical IVR	%
X15	Incremental IVR	IU·dL^−1^/IU·kg^− 1^
X16	Peak level of FVIII in operation day	% or IU·dL^−1^
X17	Operation duration	mins
X18	Transfuse blood	0 = no blood transfusion; 1 = blood transfusion
X19	Transfusion quantity of suspended red blood cells	U

## Results

### General information

Summarize all data, and distinguish them according to quantitative or qualitative data, in which the quantitative data are shown as means±standard deviation; See Table 2 for details. Qualitative information is shown in percentage; See Table 3 for details.

**Table 2:** Quantitative data (mean ± standard deviation)

Variable	Name	Mean standard deviation
Y1	Total blood loss	1221.79 ± 349.89
Y2	Intraoperative blood loss	152.33 ± 67.65
Y3	Hidden blood loss	1069.51 ± 341.99
X1	BMI	20.00 ± 2.11
X2	Age	38.72 ± 8.74
X4	Total blood volume	4342.88 ± 282.75
X8	Preoperative HB	142.92 ± 13.02
X9	Preoperative HB	107.85 ± 12.86
X10	Preoperative HCT	42.30 ± 3.94
X11	Postoperative HCT	31.92 ± 3.85
X12	FVIII peak level	118.18 ± 9.22
X13	FVIII valley level	0.90 ± 1.06
X14	Classical IVR	138.37 ± 16.00
X15	Incremental IVR	2.75 ± 0.32
X16	Peak level of FVIII in the operation day	96.70 ± 9.07
X17	Operation duration	125.53 ± 27.33
X18	FVIII dose	43.06 ± 4.17
X19	Blood transfusion quantity	0.565 ± 1.369

**Table 3:** Qualitative data

Variable	Name	Classify	Quantity	Proportion
X3	Sex	man	92	100.0%
		woman	0	0.0%
X5	TXA	No TXA	46	50.0%
		With TXA	46	50.0%
X6	FVIII prophylaxis	No FVIII prophylaxis	57	62.0%
		Low dose regimen	35	38.0%
		Medium dose regimen	0	0.0%
		High dose regimen	0	0.0%
X7	HBV	No HBV	43	46.7%
		HBV	49	53.3%
X18	Blood transfusion	No blood transfusion	77	83.7%
		blood transfusion	15	16.3%

### Multi-factor linear regression factor screening

According to the professional knowledge, we analyzed the independence of each variable and preliminarily screened out eight variables: BMI, age, incremental IVR, operation duration, TXA, HBV, FVIII prophylaxis, and FVIII peak level on the operation day. Single-factor linear regression was conducted for the above variables, and the results are shown in Table 4.

**Table 4:** Single factor linear regression result table

Variable	B value	B standard error	B standardized value	T value	*P* value
age	18.520	3.631	0.474	5.100	<0.001
BMI	30.536	16.753	0.189	1.822	0.072
incremental IVR	− 235.722	110.481	−0.219	−2.134	0.036
TXA intervention^a^	− 505.152	48.023	−0.743	−10.519	<0.001
FVIII prophylaxis^b^	− 310.673	66.189	− 0.443	−4.694	<0.001
HBV^c^	18.905	71.830	0.028	0.263	0.793
Operation duration	0.574	1.645	0.037	−0.349	0.728
Peak level of FVIII in operation day	−2.038	3.967	−0.054	−0.514	0.609

^a^Without TXA as control

^b^Without FVIII prophylaxis as control

^c^Without HBV as control

The variables with *P* values less than 0.05 were included in the multivariate linear regression analysis. There were four variables: age, incremental IVR, TXA, and FVIII prophylaxis.

### Results of multi-factor linear regression analysis

In this study, multivariate linear regression was used to predict hidden blood loss according to TXA, age, incremental IVR, and FVIII prophylaxis. It is judged that there is a linear relationship between independent variables and dependent variables by drawing partial regression scatter plots and scatter plots of student-based residual and predicted values. It has been verified that the research observations are independent of each other (Durbin-Watson test value is 1.665); By drawing the scatter diagram between the student residual and the non-standardized predicted value, it is proved that the variance of the residual is equal. The regression tolerance is greater than 0.1, and there is no multicollinearity. In the abnormal value test, there is no observed value with student deletion residual greater than three times the standard deviation, the data leverage value is less than 0.2, and there is no value with Cook distance greater than 1. P-P diagram indicates that the residual is an approximately normal distribution. The regression model is statistically significant, F = 41.469, *P* < 0.001, and adjusted *R*^*2*^ = 0.640. The influence of the four independent variables included in the model on the hidden blood loss was statistically significant (*P* < 0.05), and the specific results are shown in Table 5. The regression model is Y = 1426.303 + 8.884X_2_–395.806X_5_–130.529X_6_–164.983X_15_. (Y: hidden blood loss, X_2_: age, X_5_: TXA, X_6_: FVIII prophylaxis, X_15_: incremental IVR).

**Table 5:** Results of multivariate regression analysis

Variable	B value	B standard error	B standardized value	T value	*P* value
intercept	1426.303	226.473	–	6.298	<0.001
TXA intervention^a^	−395.806	49.281	−0.582	−8.032	<0.001
FVIII prophylaxis^b^	−130.529	47.720	−0.186	−2.735	0.008
age	8.884	2.648	0.227	3.354	<0.001
incremental IVR	−164.983	68.014	−0.154	−2.426	0.017

^a^without TXA as control

^b^without FVIII prophylaxis as control

## Discussion

This study only includes males, because that hemophilia is commonly affecting male. Therefore, we were unable to analyze the effect of sex on hidden blood loss. Zhai et al. [14] compared blood loss in HA patients with osteoarthritis patients, and showed that the total and hidden blood loss in HA group were significant higher than those in the osteoarthritis group, while there was no significant difference in visible blood loss. In our study, the total blood loss in HA patients was 1221.79 ± 349.89 mL, while the hidden blood loss was 1069.51 ± 341.99 mL.Hidden blood loss is the main cause of blood loss, so from this perspective, hidden blood loss is the focus of HA blood management. Multivariate linear regression showed that TXA, FVIII prophylaxis, age and incremental IVR were related to hidden blood loss. However, the length of operation, the peak level of FVIII during operation, HBV, and BMI did not show any correlation.

Many research [15–17] showed that TXA in TKA can effectively reduce blood loss, and the research of Huang [18] showed that TXA in TKA can also make a great hemostatic effect for HA patients. In our study, patients injected with 1gTXA before the closure of the joint cavity will reduce the hidden blood loss (b = − 395.806). TXA is a synthetic derivative of amino acid lysine. It inhibits fibrinolysis by reversibly blocking lysine binding sites on plasminogen. It also inhibits the activation of plasminogen by plasminogen activator. As the level of plasmin is lowered, the fibrinolytic activity is weakened, and fibrin is not decomposed, thus reducing bleeding [19]. Using TXA in TKA to reduce blood loss has been a common choice of clinicians, but there is no unified view on the administration mode and dosage of TXA. Sarzaeem et al. [20] compared three ways of TXA administration: intravenous injection, articular cavity injection, and drainage tube injection. The results showed that all three ways could reduce blood loss, while the intravenous injection was better than the others. A meta-analysis of Alshryda et al. [21] showed that the hemostatic effect of local injection was better than that of the intravenous route. Keyhani et al. [22] showed that local injection and intravenous injection have similar effects. In addition, although VTE is rare in HA patients, we are concerned about the increased risk of VTE in HA patients who have high postoperative levels of FVIII while using only physical prophylaxis, if TXA is administered intravenously. Therefore, we carefully choose joint cavity local injection as the TXA administration method.

Then the FVIII prophylaxis is taken into discussion. FVIII prophylaxis is considered as the main treatment of HA [23], playing an important role in preventing bleeding and arthritis. We indeed found that the hidden blood loss of patients treated with FVIII prophylaxis was lower (b = − 130.529). We suspect that FVIII prophylaxis can reduce the frequency and severity of joint bleeding effectively, thus reducing the damage degree of muscles, bones, and blood vessels, so the bleeding in these tissues will decrease after TKA. FVIII prophylaxis was advocated, and it is recommended to adopt the medium-dose regimen or the high-dose regimen [24], but it also means a huge economic burden. In the United States, the annual medical expenditure of each HA patient is 250,000 US dollars [25], which is unrealistic in developing countries. Among our patients, only 35 patients were treated with the low-dose regimen, and no patients were treated with medium and high-dose regimen. Therefore, we failed to explore the influence of different dosage schemes on TKA hidden blood loss. We suggest that every patient should make an individualized FVIII prophylaxis plan with a consideration of economic factors. And the minimum dose of FVIII prophylaxis should be maintained on the premise of ensuring the quality of treatment.

Besides, multivariate linear regression showed that age was a positive influencing factor of hidden blood loss (b = 8.884), Dong [26] and others found that the activity of chaperone-mediated autophagy will decrease with the increase of age, thus leading to the decline of the function of hematopoietic stem cells. It is more difficult for patients to self-correct anemia. Therefore, we still need to be more careful to the greater risk of bleeding for older patients, and take appropriate additional preventive measures.

Moreover, multivariate linear regression showed that the incremental IVR was the negative influencing factor of hidden blood loss (b = − 164.983). Theoretically, the dosage and frequency of FVIII infusions for each patient should be individualized according to the incremental IVR. Actually, according to the instructions of FVIII, the value of incremental IVR is usually regarded as 2 IU dL^− 1^/IU kg^− 1^. Our study also used this method to calculate the dose of FVIII. In the post-analysis, the incremental IVR of our patients was 2.75 ± 0.32 IU dL^− 1^/IU kg^− 1^. This indicates that the actual FVIII level will be higher than expected, but fortunately, it will reduce the hidden blood loss. However, the higher FVIII does not mean a better result. VTE may occur if the FVIII level is higher than the ideal level for a long time [27]. In our study, VTE was not observed, so it was impossible to evaluate the influence of high FVIII levels on VTE. All in all, the individualized difference of incremental IVR will lead to the deviation of perioperative FVIII level control. Only by calculating the infusion dose of FVIII based on the individual incremental IVR can we avoid massive hemorrhage or thrombosis.

At last, we also look into the influence of HBV on hidden blood loss. Before the emergence of FVIII products, HA patients were usually treated with plasma when bleeding, and transfusion-related hepatitis became a vital complication of hemophilia [28]. Among our patients, 49 have HBV, the proportion is as high as 53.3%, and the liver injury may affect the coagulation, so HBV must be taken into account as a factor of hidden blood loss. All of our patients had been given the formal liver-protecting and antiviral therapeusis before the operation. The operation will not be allowed if their alanine transaminase and aspertate aminotransferase are beyond the normal range. Under this premise, the influence of HBV on hidden blood loss was not statistically significant (*P* = 0.793). Three of our patients were infected with Hepatitis C virus, but they were excluded because they were simultaneously infected with HIV, and no patients were infected with Hepatitis D virus. Therefore, we only discussed the effect of HBV on hidden blood loss. AIDS is also a severe complication of hemophilia [29]; however, in our center, the patients with human immunodeficiency virus (HIV) are not as common as those with hepatitis, there are only three HA patients with HIV who have undergone TKA, and their blood loss is much higher than that of ordinary patients. Meanwhile, the perioperative management of HA patients with HIV, including the surgical plan, is more complicated [30], so it is necessary to formulate a more detailed treatment plan for HA patients with HIV. Therefore, we exclude HA patients with HIV from the study.

## Conclusion

The blood loss of TKA mainly comes from hidden blood loss, which is as high as 1069.51 ± 341.99 mL. The main influencing factors are age, TXA, FVIII prophylaxis, and incremental IVR. Elderly patients have a higher risk of blood loss, so perioperative blood management should be strengthened. TXA can effectively reduce hidden blood loss, and local injection into the articular cavity is recommended. FVIII prophylaxis can not only improve the joint condition but also reduce blood loss. It is recommended that every patient take FVIII prophylaxis. The incremental IVR affects the accuracy of perioperative FVIII level, which easily leads to serious consequences of blood loss or thrombosis. It is recommended that each patient measure the individual incremental IVR and formulate the FVIII infusion scheme, which can not only ensure the treatment quality but also reduce the economic burden greatly.

## Data Availability

Data cannot be provided due to identifying information of participants but are available from the corresponding author on reasonable request.

## Abbreviations

HA: Hemophilia A
TKA: Total knee arthroplasty
IVR: Invivo recovery
CFCS: Clotting factor concentrates
FVIII: Coagulation factorVIII
VTE: Venous thromboembolism
TXA: Tranexamic acid
AIDS: Acquired Immune Deficiency Syndrome
BMI: Body mass index
IU: International units
HCT: Hematocrit
HB: Hemoglobin
HBV: Hepatitis B virus
HIV: Human immunodeficiency virus
